# Confident but incomplete: gaze behavior and driving dynamics in deadlock situations at T-intersections

**DOI:** 10.3389/fpsyg.2026.1931680

**Published:** 2026-09-17

**Authors:** Nadine-Rebecca Strelau, Barbara Deml

**Affiliations:** Institute of Human and Industrial Engineering, Karlsruhe Institute of Technology, Karlsruhe, Germany

**Keywords:** arrival order, deadlock situation, driver behavior, gaze behavior, T-intersection

## Abstract

**Introduction:**

Deadlock situations at T-intersections pose a challenge for human drivers and automated vehicles, as formal right-of-way rules provide no clear guidance and drivers do not share a uniform interpretation of these situations. The present driving simulator study investigated how drivers approach deadlock situations before a standstill occurs and whether they perceive the situation as a conflict.

**Method:**

Participants drove through an urban environment and encountered deadlock situations from all three possible approach positions while the arrival order of the cooperating vehicles was varied. Perceived ambiguity was assessed after each encounter. Eye movements and driving parameters were analyzed in the segment between 25 m and 10 m before the intersection, and driving order was recorded.

**Result/Discussion:**

Results show that drivers generally did not perceive deadlock situations as ambiguous. Gaze analyses revealed that at least one cooperating vehicle was often not fixated during the approach, indicating that the full deadlock structure and thus the potential conflict was often not visually processed. Differences in gaze behavior and driving dynamics between drivers who proceeded first and those who stopped emerged only for the left approach. Driving order was influenced by the interaction of approach position and arrival order, with drivers from the left frequently proceeding first when the vehicle from below arrived later. Additionally, a random forest classifier provided a proof of concept for data-driven prediction of driving order based on externally observable approach-related variables. Together, these findings offer a deeper understanding of driver behavior in deadlock situations and provide recommendations for automated vehicles’ behavior aimed at preventing standstills.

## 1 Introduction

Automated vehicles (AVs) are increasingly being introduced into everyday traffic and are expected to make driving safer, more comfortable, and more efficient. However, in mixed traffic environments, successful interaction between automated and human drivers remains a fundamental challenge, particularly in complex and dynamic urban settings. Conservative and defensive behavior may be the safest strategy for AVs, yet it can also have unintended consequences. Strict compliance with traffic rules or overly cautious behavior may be unexpected by human drivers and can lead to reduced traffic flow, congestion, frustration and in some cases even contribute to accidents (Alambeigi et al., 2020; Schwarting et al., 2019).

Understanding how human drivers behave and coordinate with others is therefore crucial for designing AVs that can interact safely and efficiently. This is particularly important in ambiguous traffic situations. Deadlock situations at intersections represent an example of such ambiguity. Navigating intersections already constitutes a cognitively demanding task, and ambiguity further increases when traffic rules themselves fail to establish a clear priority. This occurs, for example, at a T-intersection regulated by right-before-left, where two vehicles intend to turn left while a third continues straight ahead (see Figure 1). Each driver must yield to another, resulting in a configuration in which none of the vehicles holds an unambiguous right of way. Consequently, traffic may come to a standstill unless drivers successfully coordinate who proceeds first.

**Figure 1:**
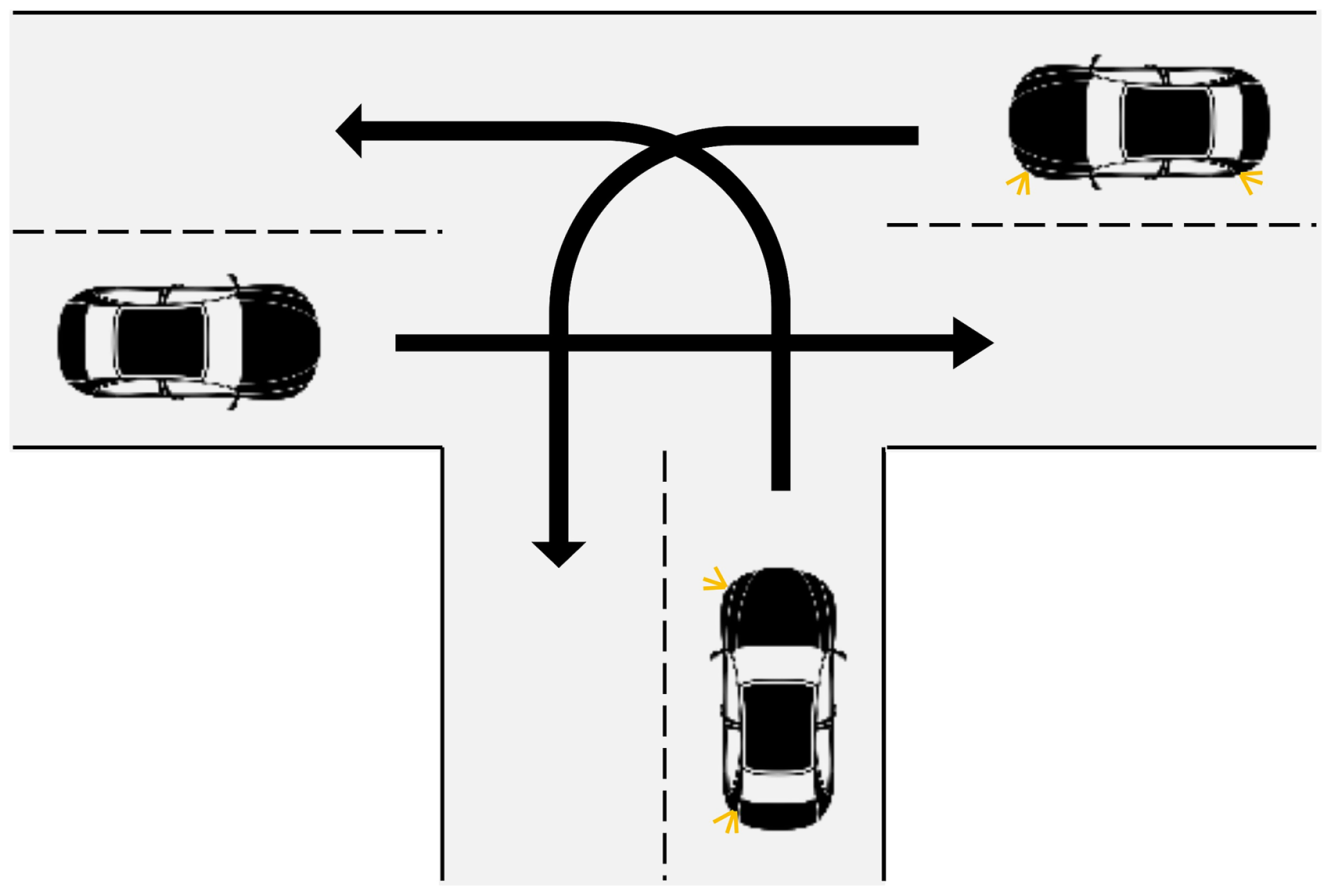
Deadlock situation at a T-intersection. Reprinted from Strelau and Deml (2025), under the terms of the CC BY 4.0 license.

Investigating how human drivers perceive and resolve such situations provides essential insights for understanding the mechanisms of coordination and for enabling automated vehicles to behave safely and efficiently without contributing to unnecessary standstills or conflicts.

## 2 Theoretical background

### 2.1 Previous research on deadlock situations

Research examining driver behavior in deadlock situations at T-intersections is still limited. Imbsweiler et al. (2018) found that implicit communication of the cooperating vehicles influences drivers’ behavior. When a cooperating vehicle shows defensive behavior such as braking, drivers will drive first, whereas offensive behavior of the cooperating vehicle leads them to stop and yield. Moreover, Imbsweiler et al. (2018) showed that, in complex deadlock situations, drivers often prefer another driver to initiate and proceed first. Building on this finding, Strelau and Deml (2025) investigated whether variations in the complexity of the T-intersection influences drivers’ behavior in deadlock scenarios. The study was based on the Task-Capability Interface (TCI) model (Fuller, 2011), which assumes that drivers adjust their behavior when the perceived difficulty of a situation exceeds their individual threshold. Task difficulty results from both task demands (i.e., environmental characteristics and other road users) and driver capability (i.e., knowledge, skill, and experience). The results showed that task demands had little to no effect on perceived difficulty or drivers’ behavior. Instead, the approach direction had a significant effect on cooperation behavior. Furthermore, the observed patterns indicated that drivers may not correctly interpret the deadlock situation, pointing to a miscalibration of driver capability rather than an effect of task demands.

A follow-up study (Strelau and Deml, 2026) therefore examined the capability component of the TCI model, focusing particularly on drivers’ understanding of traffic rules and their mental representation of the intersection. The findings revealed that many drivers hold an incorrect mental model of the deadlock situation. In approximately one quarter of all trials, the travel direction of at least one cooperation vehicle was misperceived. Moreover, the majority of participants failed to correctly identify that in this situation no vehicle has the right of way. Instead, most attributed priority to one of the other vehicles, typically the one approaching from the right or, less frequently, from the left. Across all approach positions, drivers attributed priority to a given position less frequently when they were themselves approaching from that position than when they were not. Nevertheless, the right approach was the position from which drivers most frequently reported intending to proceed first. This predominant attribution of priority to the right vehicle contradicts the main-road effect described by Björklund and Åberg (2005), who found that drivers treat the straight road as a priority road in two-vehicle intersection scenarios. In the deadlock situation this would predict priority being assigned most frequently to the left vehicle traveling straight ahead instead of the right vehicle. Drivers’ expectations about others’ behavior were also shaped by approach direction and by their own perceived understanding of the right-of-way rule. As a result, drivers’ own behavior was determined by a combination of subjective rule interpretation and expectations about others’ actions. Taken together, these studies indicate that behavior in deadlock situations is guided by individual mental models, and that a shared representation of the situation is not always given. However, it remains unclear whether stopping in these deadlock situations and letting another vehicle yield reflects uncertainty about the appropriate course of action or a confident, though potentially incorrect, assessment that another vehicle should proceed first.

### 2.2 Coordination and interaction

While the TCI model (Fuller, 2011) offers a useful account of how an individual driver regulates behavior, it does not sufficiently address interactions with other traffic participants. This limitation becomes apparent in multi-vehicle scenarios such as intersections, where each driver’s decision also depends on the anticipated and observed behavior of others. Communication and the recognition of others’ intentions therefore play a crucial role in driver interaction (Wilde, 1976). Building on this, Renner and Johansson (2006) argue that models of driver behavior should be expanded beyond the single-driver perspective to include the concept of joint action.

Joint action describes situations in which two or more individuals coordinate their behavior toward a shared goal. In such settings, what one person does depends on what others do; their actions are interdependent (Clark, 1996). Effective coordination of this kind requires a sufficient level of interpredictability, which in turn depends on common ground, the shared knowledge, beliefs, and assumptions that individuals use when interpreting each other’s actions (Klein et al., 2005). In traffic, initial common ground is established through shared knowledge of traffic rules and conventions, understanding of social norms, and awareness that intentions can be communicated (through implicit and explicit signals) (Renner and Johansson, 2006). However, in ambiguous settings such as deadlocks, this common ground is reduced when drivers differ in their understanding of rules or fail to recognize that mutual coordination is required. As a result, the basis for interpredictability, and thus joint action, is reduced.

This becomes even clearer when considering the space-sharing conflict framework, which conceptualizes interaction in traffic in spatial–temporal terms (Markkula et al., 2020). Interactions between drivers occur whenever two or more road users must negotiate the order of access to a shared region of space, referred to as the conflict space. A space-sharing conflict is present when it “[…] can be reasonably inferred that two or more road users are intending to occupy the same region of space at the same time in the near future” (Markkula et al., 2020, p. 736). Whether a driver perceives such a conflict, however, depends on their interpretation of the situation. As a result, the same situation may be perceived as a conflict by one driver but not by another. Markkula et al. (2020) therefore distinguish between obvious space-sharing conflicts, where the conflict is clear, and potential space-sharing conflicts, in which uncertainty exists because drivers do not know, for example, whether the other has noticed all relevant information or how they intend to act.

Once a conflict is perceived, drivers adjust their behavior in order to resolve it by either actively changing their behavior or acquiring new information that leads them to conclude that no conflict will occur after all. Importantly, an interaction only occurs when at least two road users adapt their behavior in response to the perceived conflict. If only one driver adjusts their behavior, or if only one of them perceives the situation as a conflict, the conditions for interaction are not fulfilled. A particularly clear case arises when one road user does not notice the other at all, for example, by not looking in their direction. In such situations, the driver lacks the necessary information to represent the situation as a conflict and therefore cannot respond interactively to the other’s presence. In deadlock situations, where drivers frequently misinterpret right-of-way and may not attend to all cooperating vehicles, it is therefore an open question whether the situation is perceived as a conflict at all. The space-sharing conflict is defined not only by spatial overlap but also temporal expectations. A conflict is only perceived when drivers anticipate that vehicles might enter the conflict zone at the same time. If one vehicle approaches the intersection later than the others, this timing may make it less clear whether the vehicles will occupy the same space at the same time. Such timing differences can therefore introduce additional uncertainty about whether a conflict is present.

### 2.3 Implications for the present study

The theoretical perspectives and empirical findings outlined above provide the basis for the present study, which aims to deepen the understanding of how drivers approach and respond to deadlock situations at T-intersections before a potential standstill occurs. A particular focus is placed on the approach phase itself: understanding what drivers attend to and how they behave during the approach, rather than once vehicles have already stopped, is crucial for enabling automated vehicles to anticipate human behavior early enough to prevent a deadlock from developing into a standstill. A central question in this context is whether drivers actually perceive the deadlock as a conflict during the approach, given that previous findings suggest that many drivers misinterpret the applicable right-of-way rules and may not attend equally to all cooperating vehicles.

To assess conflict perception, two measures are used. The first is perceived ambiguity, the subjective uncertainty about the appropriate course of action. This measure does not directly capture conflict perception, but serves as a behavioral indicator of it. A driver who perceives a space-sharing conflict should experience uncertainty about how to act, whereas a driver who has formed a clear, if potentially incorrect, interpretation of right-of-way should report low ambiguity. This allows an open question from previous work (Strelau and Deml, 2026) to be addressed empirically, where it remained unclear whether drivers stop because they are uncertain about the appropriate behavior in the situation, or whether they stop because they believe another vehicle clearly has the right of way and should proceed first. This question is particularly interesting given that even drivers who expected the other vehicles to stop often reported a high likelihood of stopping themselves (Strelau and Deml, 2026). Examining perceived ambiguity in relation to driving order can help distinguish between these interpretations.

The second indicator of conflict perception is gaze behavior. A necessary precondition for perceiving a conflict is that drivers actually perceive the relevant interaction partners and form expectations about their intentions. If one vehicle receives considerably less attention, drivers may only recognize part of the conflict, a potential conflict with one vehicle, but not the triadic conflict that arises from the three vehicles together in the deadlock situation. Previous findings (Strelau and Deml, 2026) showed that the intended travel direction of the cooperating vehicles was frequently misidentified, suggesting that the two cooperating vehicles are not attended to equally. However, whether this reflects actual differences in visual attention was not measured. Eye-tracking in the present study therefore allows this to be examined directly, assessing whether each cooperating vehicle was fixated during the approach. Together with ambiguity ratings, gaze behavior provides a more complete picture of whether and to what degree drivers perceived the deadlock as a conflict with gaze behavior capturing the perceptual basis and ambiguity the subjective interpretation of the situation.

The space-sharing conflict framework further highlights that conflict perception has a temporal dimension. A conflict is only perceived when drivers anticipate that vehicles might enter the conflict zone at the same time. The arrival order of the cooperating vehicles may therefore influence conflict perception, though the direction of this effect is not straightforward. On the one hand, a vehicle arriving noticeably later may reduce the perceived likelihood of simultaneous space occupation, making it less clear whether a conflict is present. On the other hand, given that most drivers interpret the deadlock as a situation with clear priority and assigning right-of-way to one of the cooperating vehicles, a delayed arrival of the vehicle perceived as having priority may instead destabilize this interpretation and increase uncertainty. Whether and how arrival order affects perceived ambiguity and visual attention to the delayed vehicle was therefore examined empirically in the present study. As approach position has been shown to influence both behavior and rule interpretation in deadlock situations (Strelau and Deml, 2026), all three possible approach positions to the T-intersection were also considered. Both perceived ambiguity and gaze behavior were examined as a function of approach position and arrival order, as well as in relation to whether drivers proceeded first or stopped.

Finally, the present study extends previous work by examining actual driving behavior in a simulator during the approach to the intersection, rather than self-reported intended behavior as assessed in previous studies (Strelau and Deml, 2025, 2026). While Imbsweiler (2019) also examined driving behavior at deadlock situations, the analyses were not restricted to the approach phase prior to a potential standstill, and approach position and arrival order were not systematically varied. The present study therefore provides insights in how these factors influence approach behavior before a potential stillstand occurs and the decision to proceed first or stop. Beyond describing these behavioral patterns, we further examined whether driving order can be predicted from externally observable approach-related variables before the intersection is reached, using a random forest classifier. Reliably anticipating the intentions of surrounding road user is a necessary precondition for safe driving, as it allows automated vehicles to understand their environment and coordinate their actions accordingly (Fang et al., 2024). A distinction is made between predicting a vehicle’s future trajectory, its continuous spatial path, and predicting its underlying maneuver or intention, a discrete decision such as turning, changing lanes, or, as in the present study, deciding to proceed first at the intersection, with intention often serving as an intermediate step that subsequently informs trajectory generation (Chen et al., 2025; Fang et al., 2024). Such predictions rely commonly on vehicle kinematics, prior trajectories, road and lane geometry, and information about interactions with surrounding vehicles. However, enhancing the accuracy of these approaches often requires incorporating further contextual information (Chen et al., 2025; Guo et al., 2025) and their performance in complex urban scenarios, particularly at intersections, still requires improvement (Hu et al., 2026). Yet, intersections at which right-of-way is not clearly regulated, such as the deadlock situation examined here, have received little attention in this line of research so far, even though understanding driving behavior in these ambiguous situations could improve intention prediction in such scenarios.

## 3 Methods

### 3.1 Participants

A total of 45 participants took part in the study. Thirteen of them discontinued participation due to simulator sickness, and one participant had to be excluded because of technical issues. The final sample therefore consisted of 31 participants (29 male, 2 female) with a mean age of 22.70 years (*SD* = 5.01). All participants held a valid driver’s license, on average for 5.05 years (*SD* = 4.81), and reported driving an average of 4129.84 km per year (*SD* = 4794.68).

### 3.2 Apparatus

#### 3.2.1 Driving simulator

The study was conducted with a static driving simulator (see Figure 2) which is based on a VW Golf 6 and configured as an automatic vehicle. The simulator was further equipped with a central console display in addition to the vehicle’s standard dashboard. Instead of physical side and rear-view mirrors, displays are installed to provide the corresponding view. The driving environment is projected onto a curved screen using three projectors, creating a field of view of 180 degrees. The simulation environment was created with the software SILAB 7.0®.

**Figure 2:**
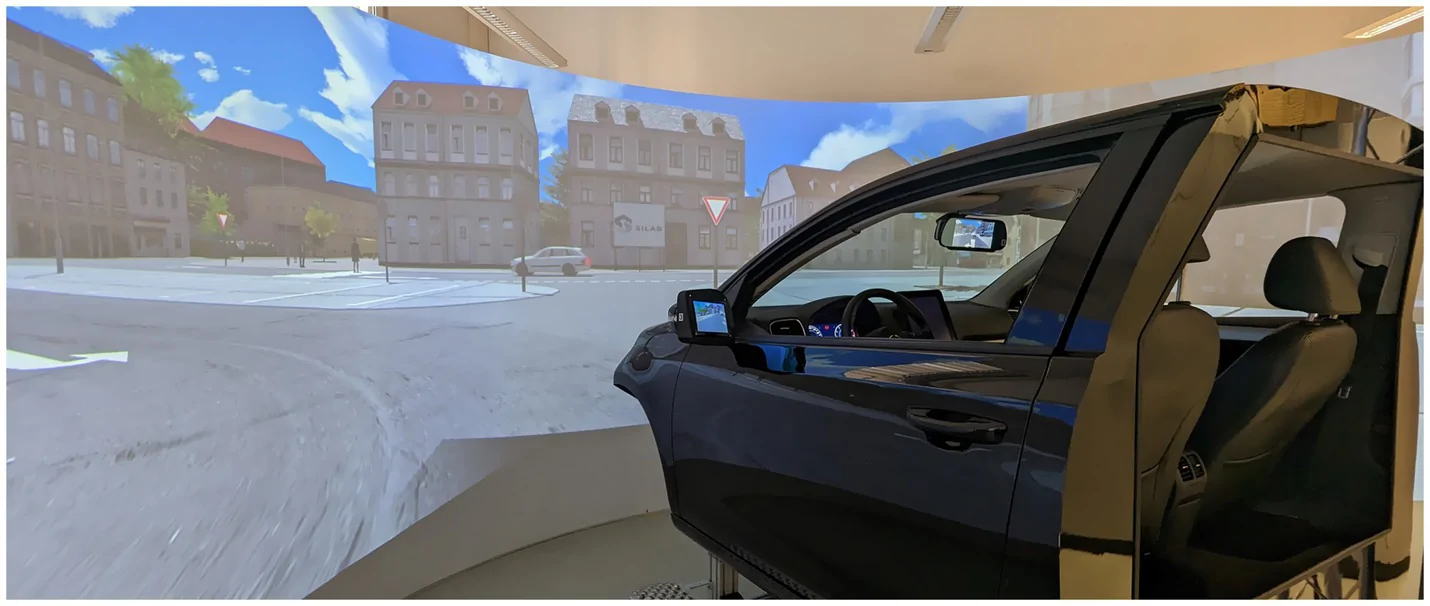
Driving simulator used in the study.

#### 3.2.2 Eyetracker

Gaze behavior was recorded using the Tobii Pro Glasses 3 wearable eye-tracker. It samples eye movements at 100 Hz and is equipped with a scene camera that captures video at 25 frames per second with a 106° diagonal field of view. The eyetracker was calibrated for each participant using a one-point calibration procedure, which the manufacturer reports to achieve an accuracy of 0.6°. For participants requiring vision correction, interchangeable lenses were used to adjust for individual prescriptions.

### 3.3 Scenario design

Participants drove through a predefined route through a suburban environment. The entire route was set in a 30 km/h zone, as indicated by traffic signs. Navigation instructions were provided with arrows displayed on the center console. The total distance was 15 km.

The intersections of interest were T-intersections without traffic signs, where the right-before-left rule applied. At these intersections, one vehicle approached from each of the two roads, creating a deadlock situation. The cooperation vehicles indicated their intended direction using turn signals. Their speed was synchronized with the participant’s speed at 10 m intervals. Twenty meters before the intersection the cooperation vehicles began decelerating at a constant rate and stopped five meters before the intersection. After remaining stationary for a period of 5 to 10 s, the vehicles continued their movement.

Between the deadlock intersections, the route included clearly regulated traffic situations (e.g., intersections without other vehicles, with traffic lights, or with a yield sign) to create a natural driving experience and to prevent participants from immediately recognizing the focus on deadlock situations.

### 3.4 Experimental design

The deadlock situation was manipulated with two independent variables: approach position and arrival order. Approach position varied across all three possible entry points of the intersection: left, right and below. Arrival order specified whether the participants and the cooperation vehicles reached the intersection simultaneously or not. It had three levels: simultaneous (the participant and both cooperation vehicles arrive at the intersection at the same time), priority-over-ego-later (the vehicle with priority relative to the ego vehicle arrives later, while the other vehicle arrives at the same time as the participant) and yielding-to-ego-later (the vehicle that has to yield to the ego vehicle arrives later, while the other vehicle arrives at the same time as the participant). In the two “later” conditions, the delayed cooperation vehicle was activated 16 m after the other vehicle, corresponding to a delay of about 2 s at a velocity of 30 km/h. This delay is smaller than the gap drivers typically accept to enter an intersection before another vehicle (Quante et al., 2021), thereby maintaining the situation as a deadlock, while creating a visibly noticeable delay. Figure 3 illustrates the three arrival order conditions schematically, using the right approach position as an example.

**Figure 3:**
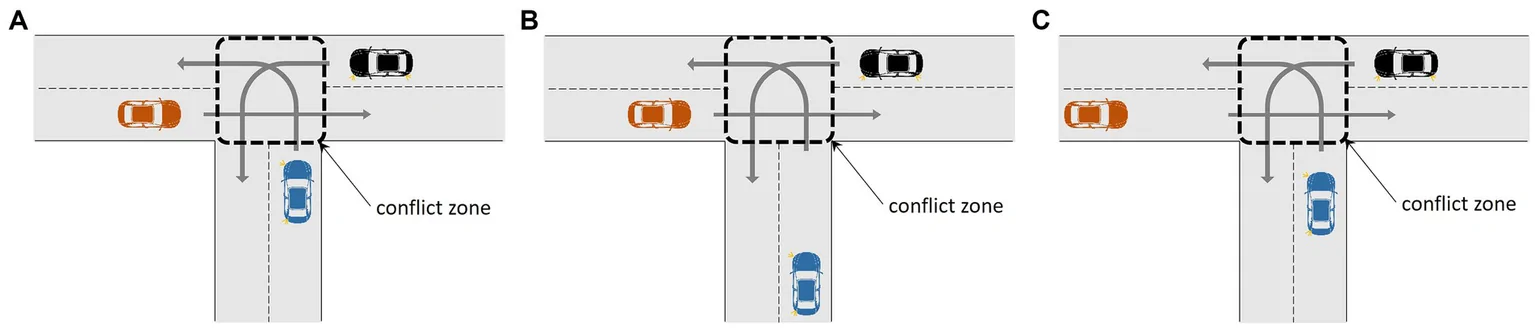
Schematic illustration of the experimental manipulation for the right approach position, showing the three arrival order conditions and the conflict zone: **(A)** simultaneous, **(B)** yielding-to-ego-later, **(C)** priority-over-ego-later. The black vehicle represents the ego perspective, the orange vehicle the cooperation vehicle with priority relative to the ego vehicle, and the blue vehicle the cooperation vehicle that has to yield to the ego vehicle. Greater distance from the intersection indicates delayed activation of the respective cooperation vehicle. The same manipulation was applied analogously for the left and below approach positions.

The manipulation of approach position and arrival order resulted in a 3 × 3 within-subject design with nine experimental conditions. Each condition was driven through twice, resulting in a total of 18 deadlock trials per participant. Trials were presented in two blocks, each containing all nine conditions once. The order of conditions was randomized independently within each block for each participant.

### 3.5 Measures

An overview of the dependent variables assessed in the study, the factors in relation to which they were examined, and the corresponding statistical approach is provided in Table 1. After each deadlock situation participants were asked two questions. They were asked to rate how ambiguous the situation appeared to them, regarding the appropriate course of action (on a scale from 1 – not ambiguous at all to 7 – very ambiguous) and how much they hesitated in deciding whether to stop or continue driving (on a scale from 1 – no hesitation at all to 7 – very strong hesitation). As the study was conducted in German, participants were asked about *Eindeutigkeit*, which corresponds to *unambiguity*. For readability and interpretability however, the construct is reported as *ambiguity* in the present paper, and the scale has been reversed accordingly. Prior to the experiment they were instructed that these questions referred to their approach to the situation, not to the period when they had already stopped or exited the situation. These questions were also asked in clearly regulated traffic situations between the deadlock scenarios.

**Table 1:** Overview of the dependent variables, their measurement, and the factors in relation to which they were examined, alongside the corresponding statistical approach.

Dependent variable (measurement)	Examined in relation to	Statistical approach
Driving order (proceeded first vs. stopped)	Approach position, arrival order	Mixed logistic regression with random intercept
Perceived ambiguity and hesitation (rated after each trial)	Approach position, arrival order	Ordinal mixed regression with random intercept
Gaze behavior (AOI fixation (fixated vs. not fixated/not visible) in the segment 25–10 m before the conflict zone)	Approach position, arrival order, driving order	Mixed logistic regression with random intercept
Continuous driving parameters (Mean speed, speed variance and acceleration in the segment 25–10 m before the conflict zone)	Approach position, arrival order, driving order	Linear mixed models with random intercept
Driving order^1^ (proceeded first vs. stopped)	Approach position, distances to the cooperation vehicles, speed, acceleration, lateral position across multiple segments (50–10 m before the conflict zone)	Random forest classifier

1 Driving order was analyzed both as an outcome explained by the experimental factors (approach position, arrival order) and, separately, as an outcome predicted from externally observable approach-related variables using a random forest classifier.

In addition to these subjective measures, driving parameters were recorded, including speed, acceleration, and lateral position relative to the lane center, as well as driving order, i.e., whether the participant proceeded first through the intersection. Only trials in which participants did not stop beforehand were classified as driving first. Trials in which participants stopped were counted as not driving first, regardless of whether they then proceeded before or after the cooperation vehicles. Once participants had stopped, the subsequent order in which the vehicles crossed the intersection may no longer have reflected the initial decision made when approaching the intersection, but rather a decision made after the standstill had already occurred. This behavior could have been influenced not only by the participant’s own intentions but also by the resumption behavior of the cooperation vehicles. After stopping, the cooperation vehicles resumed movement after a duration that varied between 5 and 10 s. Therefore, participants who had stopped could have proceeded before or after the cooperation vehicles for various reasons. For example, they may have decided to initiate movement because they no longer wanted to wait. Depending on the timing of the cooperation vehicles’ resumed movement, this same decision could have resulted in the participant either proceeding before the cooperation vehicle or, when a cooperation vehicle coincidentally began moving at the same moment, letting it proceed first instead. Alternatively, participants may simply have continued waiting until another vehicle proceeded first. As the present study focuses on driving behavior during the approach to the intersection, before a potential standstill occurred, trials in which participants stopped were therefore classified uniformly as not driving first, regardless of the subsequent driving order. This approach was chosen to ensure that driving order reflected proactive decisions made during the approach, rather than behavior that emerged only after a standstill and may have been partly influenced by the timing of the cooperation vehicles’ resuming movement.

For the eyetracking data, analysis focused on whether the two cooperation vehicles were within the participant’s visual field and whether they were fixated or not fixated. Fixations were counted if they had a minimum duration of 60 ms, which is the default value of the Tobii I-VT filter (Olsen and Matos, 2012). While fixation duration thresholds vary considerably across studies, several driving simulator studies have applied a threshold of 60 ms (e.g., Fuchs et al., 2026; Ulahannan et al., 2022; Wang et al., 2021). For both the driving parameter data (i.e., speed and acceleration) and the eye-tracking data, the analysis focused on the range between 25 and 10 m before the conflict zone (see Figure 3). The lower bound of 25 m was chosen because this marks the beginning of the cooperation zone, in which interaction between drivers takes place (Imbsweiler et al., 2016). The same distance has also been used as the starting point for analyzing driving and gaze behavior in previous research on deadlock situations (Imbsweiler, 2019), supporting its use as a starting point in the present study. The upper bound of 10 m was selected because most drivers in the present study had not yet come to a stop at this distance. As the present study aimed to examine driving behavior specifically during the approach phase, before a potential standstill occurred, restricting the analysis to this range ensured that the observed driving and gaze patterns reflected approach behavior rather than behavior that occurred only after participants had already stopped. Using a fixed distance range, rather than a range ending individually at each participant’s stopping point, further ensured that the same spatial segment was analyzed across all participants and trials.

### 3.6 Procedure

At the beginning of the study, participants were informed about the general procedure of the experiment. They were informed that the study investigated driving behavior in general, without mentioning that deadlock situations were the focus. Then participants completed a practice drive in the driving simulator where they could drive freely for 5 min in a city to become familiar with the simulator and the vehicle controls. After the practice drive, the experimental procedure was explained. Participants were instructed to follow the navigation arrows and were informed that the entire city was a 30 km/h zone. They were asked to drive as they would in real life. The two questions regarding perceived ambiguity and hesitation were explained. Next, the eye-tracker was calibrated. After completing the experimental drive participants filled out a demographic questionnaire.

### 3.7 Data analysis

To analyze the data the software R 4.5.1 was used with packages rstatix, ggplot2, dplyr, ordinal, RVAideMemoire, emmeans, lme4, lmerTest, MuMIn, ROSE and caret. Tobii Pro Lab 24.21 software was used to process the eye tracking data.

A total of 33 intersection trials were excluded from the analysis because at least one of the cooperating vehicles was activated incorrectly. Out of the 558 intersections initially completed by participants, 525 trials were included in the final analysis. For the analysis of the eye-tracking data, four participants were excluded because the eye-tracker calibration failed. For the remaining participants, data quality was high with a mean percentage of valid gaze samples of 98% (*SD* = 1.3). Consequently, 456 trials remained for the eye-tracking analysis. The areas of interest (AOIs) for both cooperation vehicles were manually annotated by one researcher. The AOIs were defined as rectangles drawn around each vehicle, leaving a small margin to ensure that fixations near the vehicle’s boundary were also captured. As the apparent size of the vehicles changed with their distance to the participant, AOI size was adjusted accordingly for each vehicle position. Because annotation was conducted by the same researcher throughout, the same general procedure was applied consistently across all vehicles and trials, thus supporting the comparability of the data.

Across all analyses (except the random forest classifier), the repeated-measures structure of the data was accounted for through mixed-effects models with random intercepts for participants. The specific model family was chosen according to the scale of the respective dependent variable: mixed logistic regression for binary outcomes (driving order, gaze behavior), ordinal mixed regression for ordinal ratings (ambiguity, hesitation), and linear mixed models for continuous driving parameters (speed, acceleration). Where significant main effects with more than two levels were found, pairwise post-hoc comparisons were conducted using Tukey’s method to control for multiple comparisons. Multicollinearity was assessed for all models including more than one predictor using generalized variance inflation factors. Across all models, GVIF^(1/2df) values ranged from 1.43 to 2.81, indicating no substantial multicollinearity among predictors. Homoscedasticity was assessed by visually inspecting plots of residuals against fitted values for the linear mixed models. No systematic patterns indicative of heteroscedasticity were observed. For the random forest classifier used for predicting driving order, the repeated-measures structure was instead addressed by splitting the data into training and test sets at the participant level.

## 4 Results

### 4.1 Effect of approach position and arrival order on driving first

In total, participants drove through the intersection first without stopping beforehand in 17% of the trials. An additional 11% of trials involved participants who stopped and then drove through the intersection first. In the remaining 72% of trials, participants stopped and let one or both cooperation vehicles proceed before crossing the intersection themselves. To investigate the effects of approach position and arrival order on the likelihood of driving first without stopping beforehand, logistic regression analyses were conducted. To account for the repeated-measures design, a mixed-effects model with random intercepts for participants was used. The model included approach position, arrival order and their interaction as fixed effects. The interaction between approach position and arrival order was significant (*χ^2^*(4) = 19.98, *p* < 0.001). Marginal and conditional R^2^ for this model were 0.25 and 0.49, respectively.

To assess potential order or repetition effects, an additional model including the repetition block (i.e., whether a trial occurred during participants’ first or second pass through all nine intersections) as a fixed effect was compared to the reported model. A likelihood ratio test indicated that including the repetition block did not significantly improve the model fit (*χ^2^*(1) = 1.97, *p* = 0.161), and the results of the original model without this term are therefore reported.

Figure 4 presents the percentage of trials in which participants drove through the intersection first without stopping beforehand, for each combination of approach position and arrival order. Pairwise comparisons (Tukey adjusted) of the significant interaction revealed that arrival order did not significantly affect the likelihood of driving first when approaching from the right or from below. In contrast, when approaching the intersection from the left, participants were significantly more likely to drive first if the cooperation vehicle with priority relative to the ego arrived later compared to when the cooperation vehicle required to yield to the ego arrived later (OR = 15.06, 95% CI [4.33, 52.35], *p* < 0.001) or both arrived simultaneously (OR = 21.17, 95% CI [5.50, 81.60], *p* < 0.001). Regarding approach position, participants were more likely to drive first when approaching from the right compared with approaching from below, when the cooperation vehicles arrived simultaneously (OR = 6.47, 95% CI [1.24, 33.82], *p* = 0.022) or the vehicle required to yield to the ego arrived later (OR = 12.24, 95% CI [1.80, 38.40], *p* = 0.007). Significant differences between the approach from the right and left, as well as between approaches from below and left were found only when the cooperation vehicle with priority relative to the ego arrived later (right vs. left: OR = 0.17, 95% CI [0.06, 0.54], *p* = 0.001, below vs. left: OR = 0.01, 95% CI [0.00, 0.10], *p* < 0.001).

**Figure 4:**
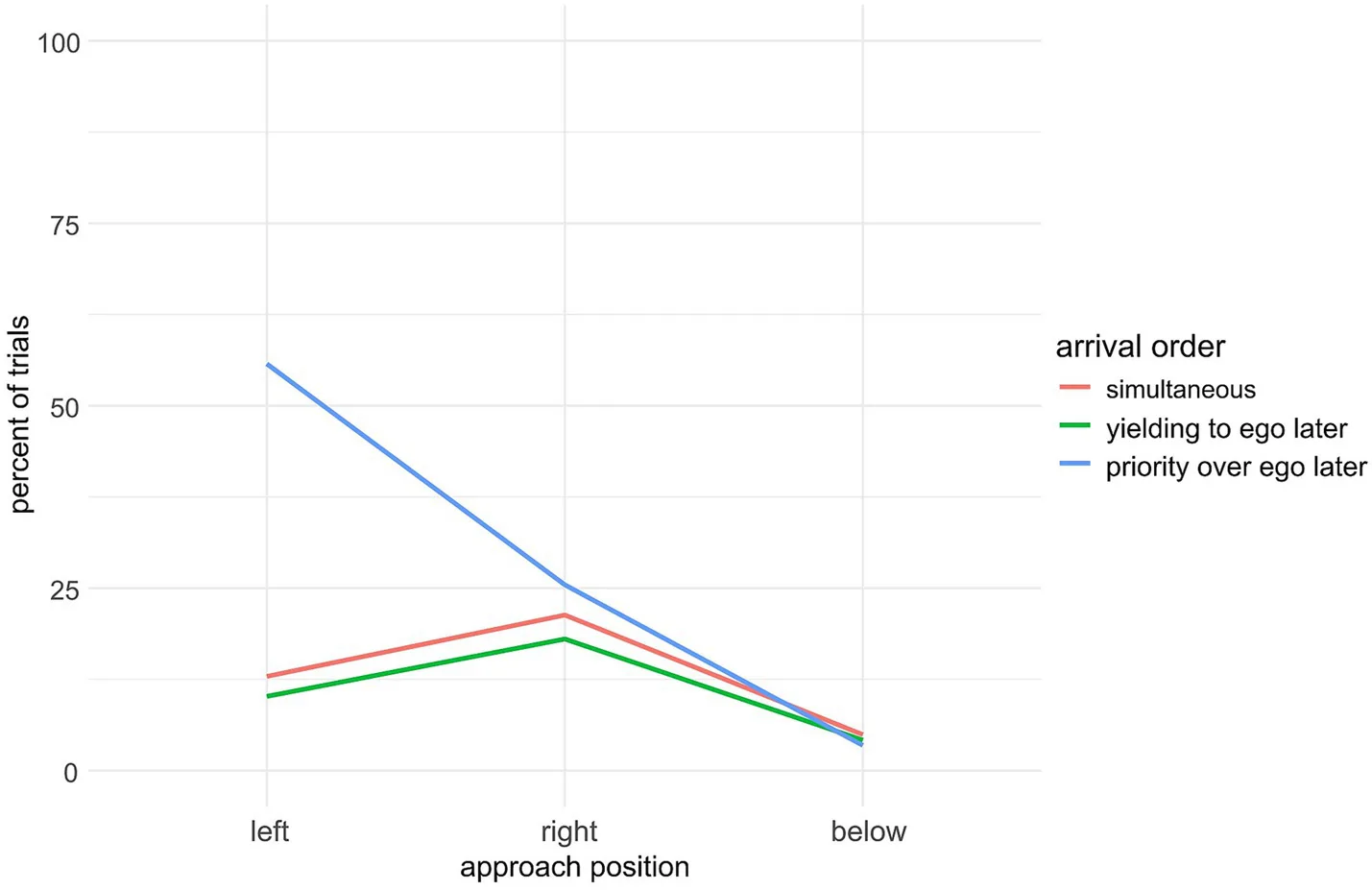
Percentage of trials in which participants drove through the intersection first without stopping beforehand depending on approach position and arrival order.

### 4.2 Ambiguity and hesitation

#### 4.2.1 Approach position and arrival order

To assess the influence of the approach position and arrival order on the perceived ambiguity and hesitation, ordinal regression models with random intercepts for participants were calculated. For both variables, null-models including a random intercept provided a significantly better fit than models without one, which supports the use of random intercept models to account for the within-subject design. The proportional odds assumption was tested using the model without random effects, as this test was not implemented for cumulative link mixed models in the used package. For both the ambiguity and hesitation model, no evidence of a violation of the assumption was found.

The arrival order had no significant effect on perceived ambiguity of the situation (*χ^2^*(2) = 0.378, *p* = 0.829) or hesitation (*χ^2^*(2) = 0.996, *p* = 0.608). The descriptive statistics are presented in Table 2. Approach position had no effect on hesitation (χ^2^(2) = 0.996, *p* = 0.608), but significantly affected ambiguity (*χ^2^*(2) = 7.104, *p* = 0.029), with approaches from below rated as less ambiguous than those from left (OR = 0.59, 95% CI [0.36, 0.95], *p* < 0.001). However, pseudo R-squared values indicate that the model explained only a small proportion of the variance (Nagelkerke’s *R^2^* = 0.030).

**Table 2:** Mean values (and standard deviation) for ambiguity and hesitation for the approach positions to the intersection and arrival order.

Measure	Approach position			Arrival order		
	Left	Right	Below	Simultaneous	Yielding-to-ego later	Priority-over-ego later
Ambiguity	3.12 (1.83)	3.03 (1.90)	2.82 (1.79)	3.08 (1.89)	2.95 (1.87)	2.94 (1.78)
Hesitation	2.48 (1.52)	2.55 (1.63)	2.27 (1.53)	2.55 (1.61)	2.43 (1.58)	2.32 (1.49)

Ambiguity and hesitation were measured on a 7-point-likert scale from 1 to 7.

Additional models including the repetition block as fixed effects were fitted to examine potential order effects. For both ambiguity and hesitation, the first run through the intersections was rated significantly higher than the second run (ambiguity: *M* = 3.20 (*SD* = 1.85) vs. *M* = 2.79 (*SD* = 1.82), *χ^2^*(1) = 17.047, *p* < 0.001; hesitation: *M* = 2.73 (*SD* = 1.73) vs. *M* = 2.14 (*SD* = 1.30), *χ^2^*(1) = 25.341, *p* < 0.001).

#### 4.2.2 Driving order

Participants who drove through the intersection first without stopping beforehand rated the ambiguity of the situation on average at 2.87 (*SD* = 1.53) and their hesitation at 2.34 (*SD* = 1.33). Participants who stopped, regardless of whether they subsequently crossed the intersection before or after the cooperation vehicles, rated ambiguity on average at 3.02 (*SD* = 1.91) and hesitation at 2.45 (*SD* = 1.60). Ratings of both ambiguity and hesitation did not differ significantly between participants who drove first and those who stopped (ambiguity: *χ^2^*(1) = 0.388, *p* = 0.534; hesitation: *χ^2^*(1) = 0.991, *p* = 0.320).

### 4.3 Gaze behavior

For the statistical analyses, gaze behavior was coded as a binary variable (fixated vs. not fixated, with the latter category including both cases in which the vehicle was visible but not fixated and cases in which it was not visible). Descriptive results are nonetheless reported separately for all three categories (fixated, not fixated, not visible), as this distinction may provide additional information on the underlying reasons for the absence of fixation. Gaze behavior was analyzed using mixed logistic regression models with random intercepts for participants to account for the repeated-measures design. Pairwise *post-hoc* comparisons were conducted using Tukey’s method to control for multiple comparisons.

#### 4.3.1 Approach position and arrival order

Across all trials, in 44% of the cases one of the cooperation vehicles was either not visible or not fixated within 25–10 m before the conflict zone. Figure 5 shows the distribution of trials in which the vehicles were not visible, visible but not fixated, or fixated, separately for the three approach positions for both cooperation vehicles. Table 3 shows the corresponding distribution separately for the three arrival order conditions.

**Figure 5:**
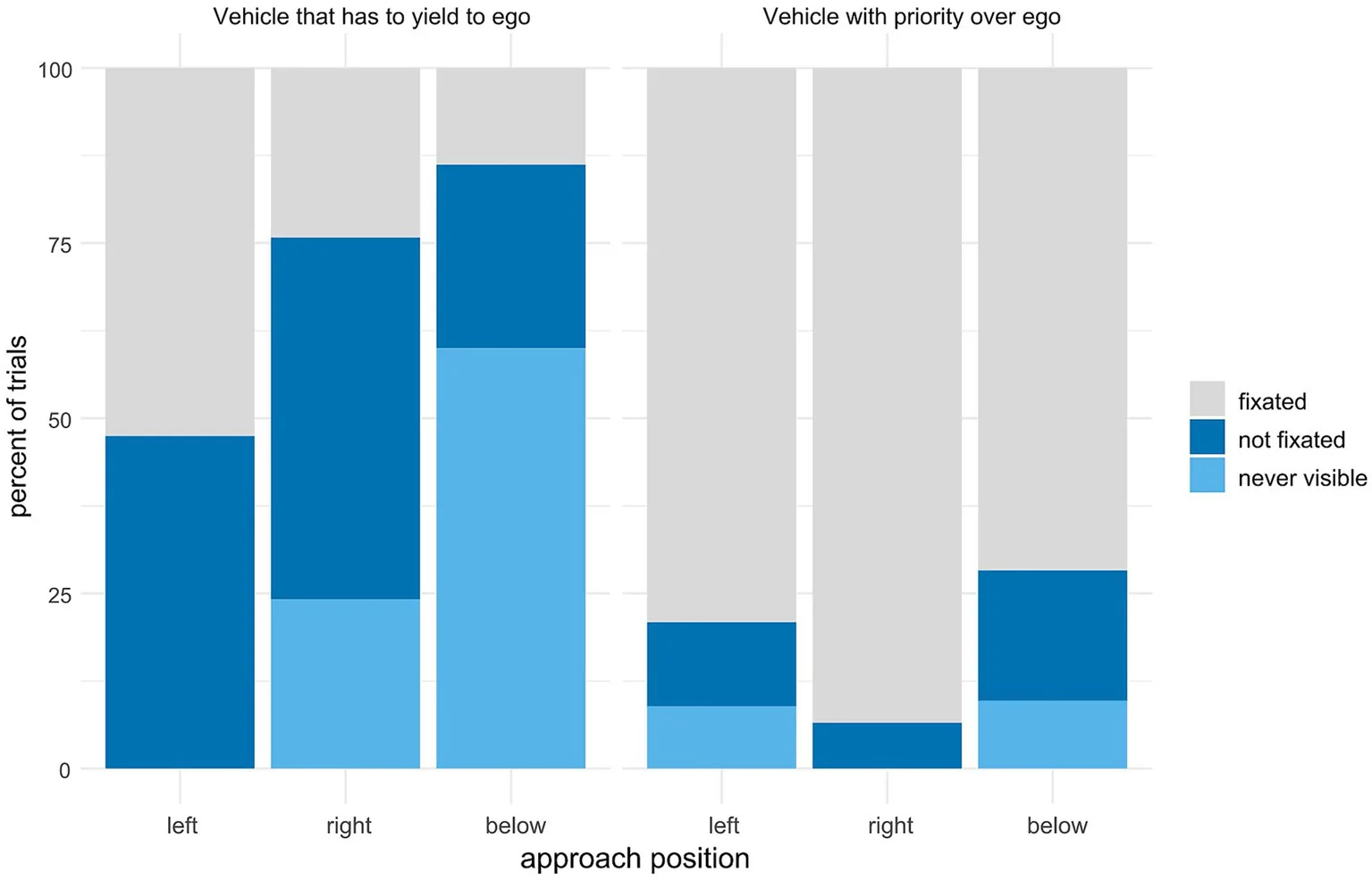
Gaze behavior toward the two cooperation vehicles across the three approach positions within 25–10 m before the conflict zone.

**Table 3:** Gaze behavior 25–10 m before the conflict zone depending on the role of the cooperating vehicle (yielding or holding priority relative to the ego) and arrival order.

Cooperation vehicle	Arrival order	Gaze behavior		
		Vehicle fixated	Vehicle not fixated	Vehicle not visible
Vehicle with priority relative to the ego vehicle	Simultaneous	84.4%	15.6%	0%
	Yielding-to-ego-later	88.9%	9.7%	1.4%
	Priority-over-ego-later	71.7%	11.2%	17.1%
Vehicle that has to yield to the ego vehicle	Simultaneous	31.2%	51.2%	17.5%
	Yielding-to-ego-later	19.4%	29.9%	50.7%
	Priority-over-ego-later	40.8%	44.1%	15.1%

The vehicle with priority relative to the ego was significantly more often fixated than the vehicle that has to yield to the ego, *χ^2^*(1) = 209.17, *p* < 0.001, OR = 11.50, 95% CI [8.26, 16.01], *R^2^_m_ = 0.29, R^2^_c_ = 0.35.* For both cooperation vehicles, approach position and arrival order had significant effects on gaze behavior (priority vehicle: approach position: *χ^2^*(2) = 6.80, *p* = 0.033, arrival order: *χ^2^*(2) = 14.89, *p* < 0.001; yielding vehicle: approach position: *χ^2^*(2) = 20.89, *p* < 0.001, arrival order: *χ^2^*(2) = 9.26, *p* = 0.010). The interaction between approach position and arrival order was not significant for either vehicle (priority vehicle: *χ^2^*(4) = 4.22, *p* = 0.377; yielding vehicle: *χ^2^*(4) = 0.38, *p* = 0.984). Marginal and conditional *R^2^* were 0.20 and 0.35, respectively, for the model of the vehicle with priority relative to the ego, and 0.23 and 0.34, respectively, for the model of the vehicle that has to yield to the ego. Additional models including the repetition block as fixed effect were fitted to examine potential order or repetition effects. For both vehicles, the model fit did not improve significantly when the repetition block was included (priority vehicle: *χ^2^*(1) = 0.01, *p* = 0.925; yielding vehicle: *χ^2^*(1) = 0.40, *p* = 0.982).

For the vehicle with priority relative to the ego, post-hoc comparisons showed that fixation was significantly more likely when approaching from the right compared to the left (OR = 3.67, 95% CI [1.35, 9.98], *p* = 0.007) or from below (OR = 6.84, 95% CI [2.62, 17.83], *p* < 0.001). The comparison between left and below approaches was not significant (OR = 1.86, 95% CI [0.90, 3.88], *p* = 0.114). For the vehicle that has to yield to the ego, fixation was significantly more likely when approaching from the left than from the right (OR = 4.26, 95% CI [2.22, 8.19], *p* < 0.0001) or from below (OR = 9.11, 95% CI [4.19, 19.81], *p* < 0.0001). The comparison between right and below approaches did not reach significance (OR = 2.14, 95% CI [0.95, 4.81], *p* = 0.073).

Regarding arrival order, post-hoc comparisons showed that fixation was significantly less likely for the vehicle with priority relative to the ego when this vehicle itself arrived later than in the simultaneous condition (OR = 0.43, 95% CI [0.19, 0.96], *p* = 0.037) or the yielding-later condition (OR = 0.28, 95% CI [0.11, 0.69], *p* = 0.003). The comparison between the simultaneous and yielding-later conditions was not significant (OR = 0.64, 95% CI [0.24, 1.70], *p* = 0.532). Similarly, for the vehicle that has to yield to the ego, fixation was significantly less likely when this vehicle itself arrived later compared to the simultaneous condition (OR = 0.44, 95% CI [0.20, 0.98], *p* = 0.041) or the priority-later condition (OR = 0.26, 95% CI [0.12, 0.57], *p* < 0.001). The comparison between the simultaneous and priority-later conditions was not significant (OR = 0.59, 95% CI [0.31, 1.13], *p* = 0.140).

#### 4.3.2 Driving order

Gaze behavior within the 25–10 m range before the conflict zone for drivers who proceeded first without stopping beforehand compared to those who stopped (regardless of whether they subsequently crossed the intersection before or after the cooperation vehicles) is shown in Figure 6 for approaches from the right and in Figure 7 for approaches from the left. For approaches from the right, gaze behavior did not significantly differ between drivers who proceeded first and those who stopped for the vehicle that has to yield to the ego (*χ^2^*(1) = 2.84, *p* = 0.092, OR = 2.49, 95% CI [0.86, 7.19]). For the vehicle with priority relative to the ego, a mixed logistic regression model could not be estimated due to complete separation: all participants who proceeded first fixated this vehicle, compared to 92% of trials among those who stopped. In contrast, for approaches from the left, gaze behavior differed significantly between drivers who proceeded first and those who stopped, for both the vehicle with priority relative to the ego (χ^2^(1) = 17.82, *p* < 0.001) and the vehicle that has to yield to the ego (χ^2^(1) = 14.37, *p* < 0.001). Drivers who proceeded first were significantly less likely to fixate the priority vehicle than drivers who stopped (OR = 0.09, 95% CI [0.03, 0.28]), whereas they were significantly more likely to fixate the yielding vehicle than drivers who stopped (OR = 5.53, 95% CI [2.28, 13.39]). Marginal and conditional R^2^ were 0.23 and 0.34, respectively, for the priority vehicle model, and 0.14 and 0.20, respectively, for the yielding vehicle model.

**Figure 6:**
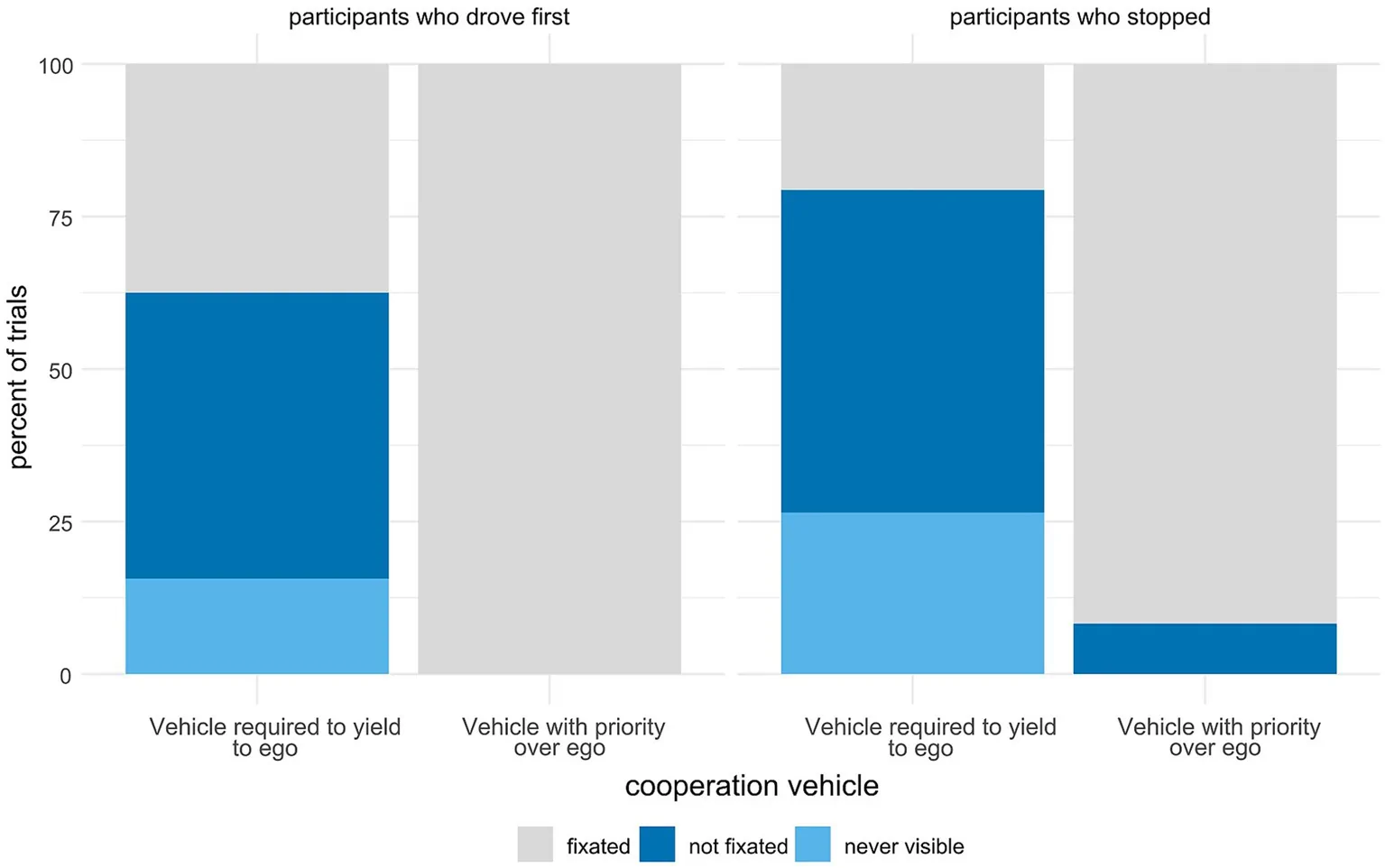
Gaze behavior 25–10 m before the conflict zone for drivers who proceeded first through the intersection and those who stopped, for both cooperation vehicles for approaches from the right.

**Figure 7:**
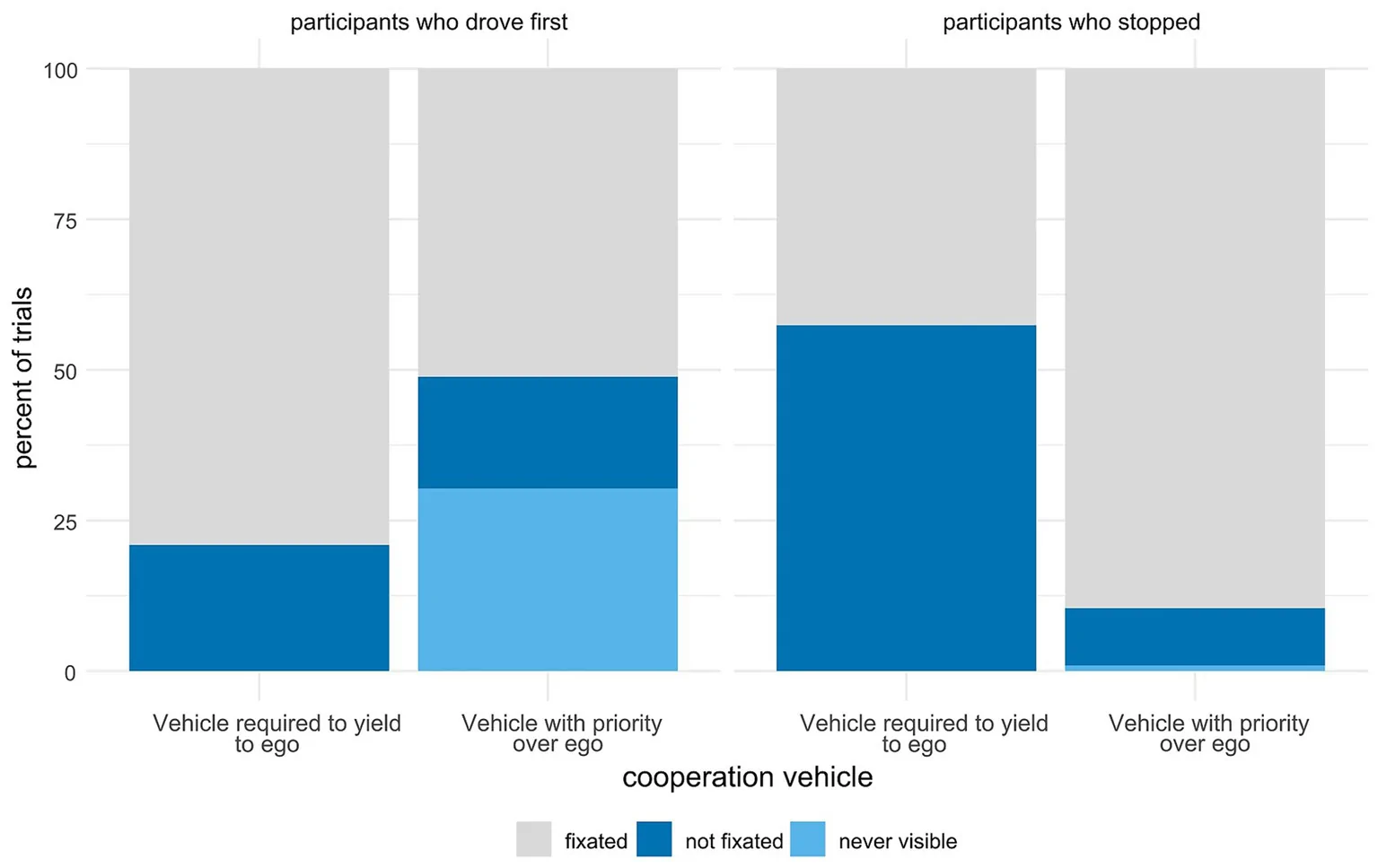
Gaze behavior 25–10 m before the conflict zone for drivers who proceeded first through the intersection and those who stopped, for both cooperation vehicles for approaches from the left.

### 4.4 Driving behavior

Differences in driving behavior, specifically speed and acceleration, were analyzed with respect to arrival order and whether participants drove first without stopping beforehand or stopped at the intersection (regardless of whether they subsequently crossed the intersection before or after the cooperation vehicles). Due to the limited number of participants driving first when approaching from below, only approaches from the left and right are included in the following analyses. The speed profiles for the different arrival orders, separated by trials in which participants drove first versus stopped, are shown in Figures 8, 9.

**Figure 8:**
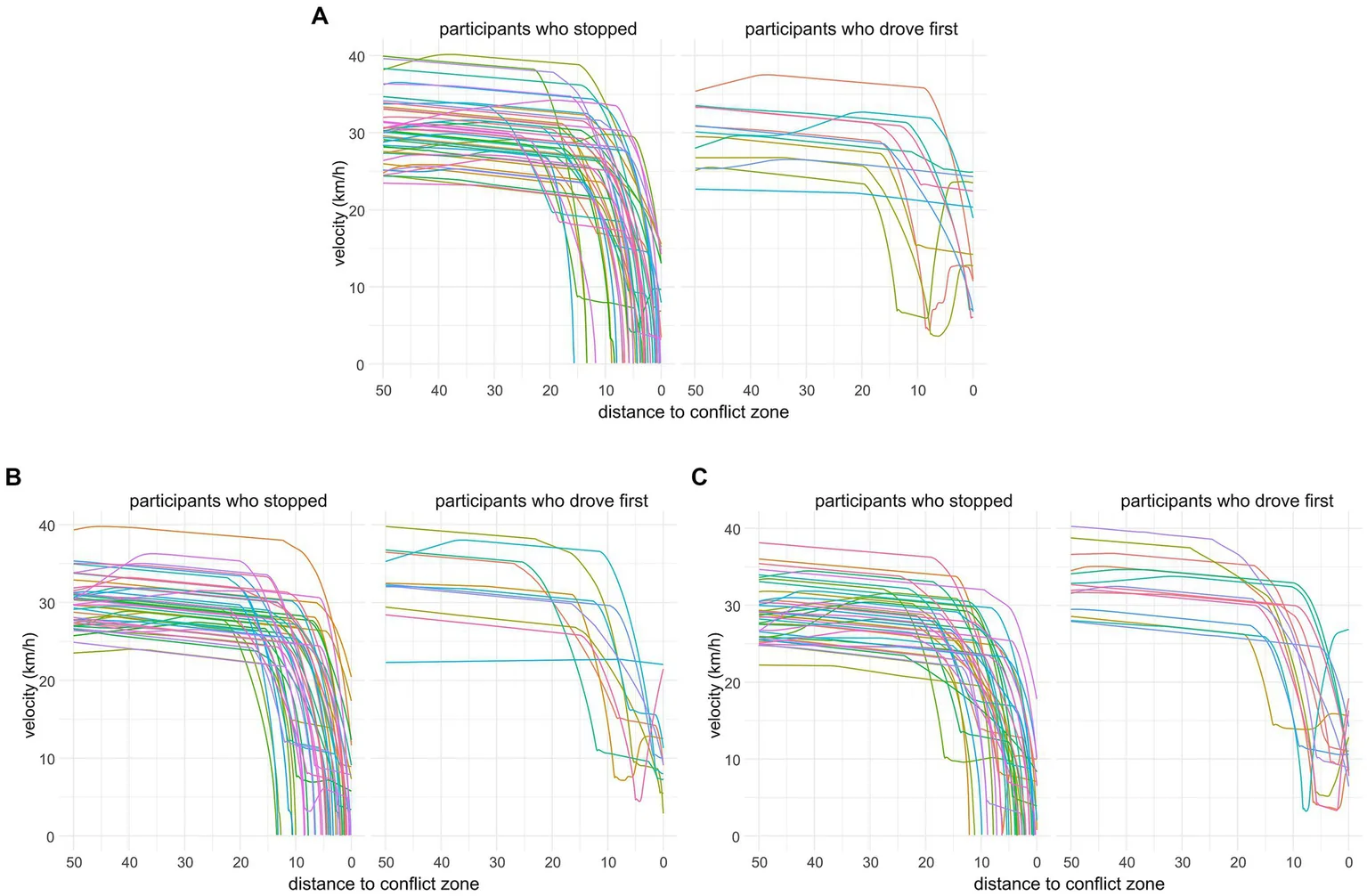
Speed profiles 50–0 m before the conflict zone for approaches from the right for trials where participants drove first and stopped for **(A)** Arrival order simultaneous, **(B)** Arrival order yielding later and **(C)** Arrival order priority later.

**Figure 9:**
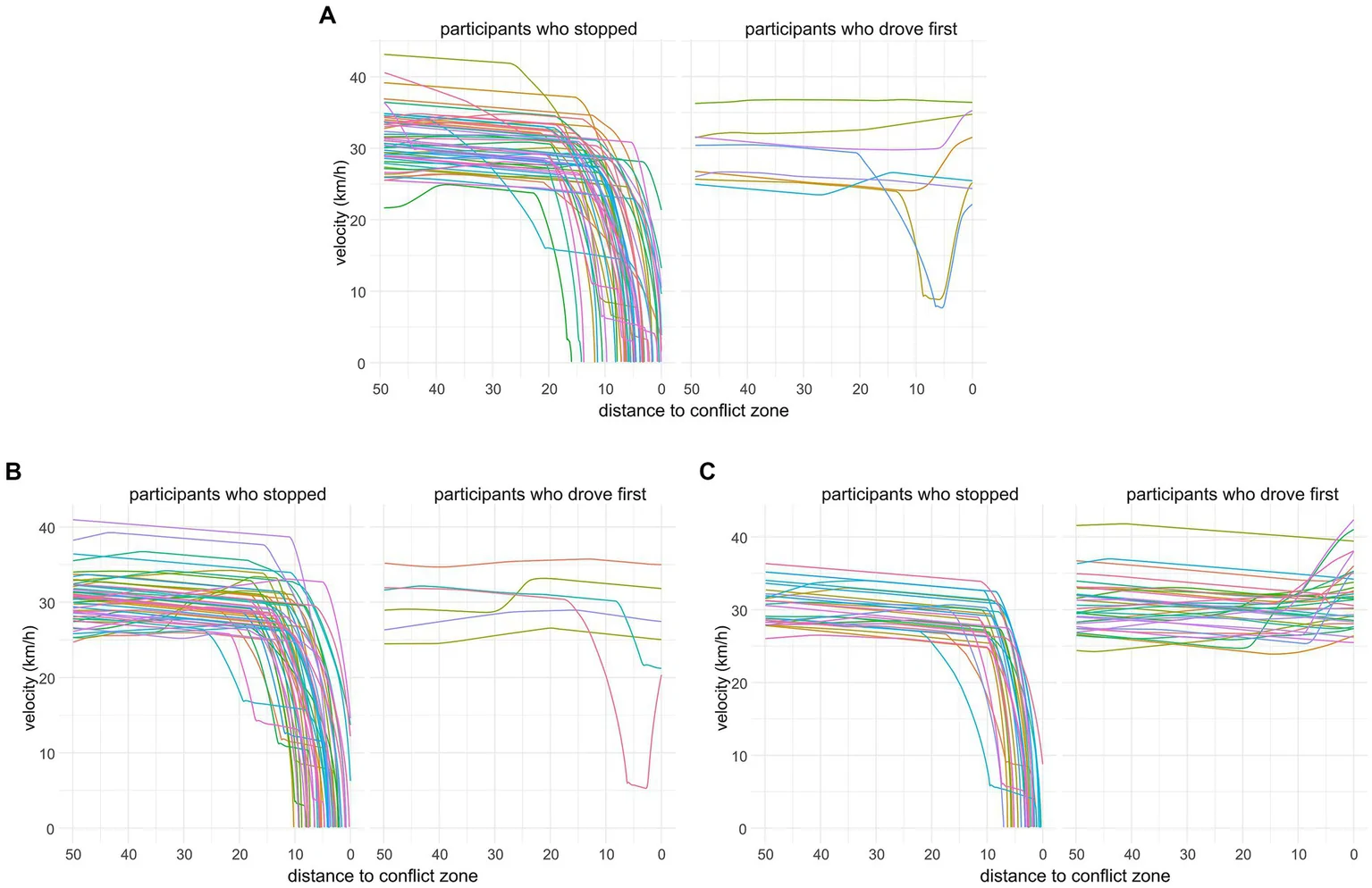
Speed profiles 50–0 m before the conflict zone for approaches from the left for trials where participants drove first and stopped for **(A)** arrival order simultaneous, **(B)** arrival order yielding later, and **(C)** arrival order priority later.

For subsequent analyses, the focus was on the approach segment from 25 m to 10 m before the intersection. For all three variables, mean speed, variance of speed and acceleration, linear mixed models with a random intercept for participants were calculated. As the residuals showed moderate deviations from normality, all variables were log-transformed. For acceleration, a constant was added prior to transformation to account for negative values. After the transformations, the residuals showed sufficient approximation to normality.

When approaching from the right, mean speed (*F*(1, 169.52) = 0.09, *p* = 0.768), variance of speed (*F*(1, 164.31) = 0.01, *p* = 0.919), and acceleration (*F*(1, 159.10) = 0.01, *p* = 0.967) did not differ significantly between trials in which participants drove first and those in which they stopped. Arrival order also had no significant effect on mean speed (*F*(2, 141.14) = 0.08, *p* = 0.921), variance of speed (*F*(2, 143.72) = 0.67, *p* = 0.512) and acceleration (*F*(2, 143.22) = 0.18, *p* = 0.836) for this approach position.

When approaching from the left, mean speed did not differ significantly between trials where participants drove first compared to those who stopped (*F*(1, 175.99) = 2.33, *p* = 0.129). However, participants who drove first showed significantly lower variance of speed (*F*(1, 176.00) = 20.42, *p* < 0.001) and significantly less deceleration (*F*(1, 170.23) = 12.11, *p* < 0.001) compared to those who stopped. Arrival order had no significant effect on mean speed (*F*(2, 162.23) = 2.76, *p* = 0.066), but significantly influenced variance of speed (*F*(2, 162.13) = 8.81, *p* < 0.001) and acceleration (*F*(2, 166.98) = 6.11, *p* = 0.003). The model for variance of speed had a marginal R^2^ of 0.31 and a conditional R^2^ of 0.46. The model for acceleration had a marginal R^2^ of 0.24 and a conditional R^2^ of 0.33. Tukey-corrected post-hoc tests revealed that variance of speed was significantly lower in the priority-over-ego-later condition than in the simultaneous condition (*p* < 0.001, *d* = 0.90) and yielding-to-ego-later condition (*p* < 0.001, *d* = 0.77). Deceleration was also significantly lower in the priority-over-ego-later condition compared to the other two conditions (*p* = 0.007, *d* = 0.74 and *p* = 0.025, *d* = 0.62, respectively). To examine whether the effect of arrival order on speed variance and acceleration is attributed to its confounding with driving order, an additional model was computed including only trials in which participants stopped. Arrival order remained significant for speed variance (*F*(2, 110.18) = 14.83, *p* < 0.001) and acceleration (*F*(2, 111.53) = 8.13, *p* < 0.001), with the priority-over-ego-later condition showing significantly less speed variance and deceleration than the other two conditions (variance of speed: *p* < 0.001, *d* = 1.26 and *p* < 0.001, *d* = 1.17; acceleration: *p* < 0.001, *d* = 0.97 and *p* = 0.013, *d* = 0.71). The model for variance of speed had a marginal R^2^ of 0.15 and a conditional R^2^ of 0.37. The model for acceleration had a marginal R^2^ of 0.10 and a conditional R^2^ of 0.24.

### 4.5 Predicting the driving order

To predict whether drivers proceeded first without stopping beforehand or stopped at the intersection (regardless of whether they subsequently crossed the intersection before or after the cooperation vehicles), random forest models were trained. Random forests are ensemble learning algorithms that can be used for classification tasks and are particularly suited for modeling nonlinear relationships between predictors. They have been successfully applied in various research contexts, including the prediction of driving behavior (Ward and Folkesson, 2015).

A total of 51 predictors were initially available. These included the approach position, turning direction, and the distance to both cooperation vehicles at 25 m and 10 m before the conflict zone. In addition, mean speed, speed variance, maximum and minimum speed, mean acceleration, maximum and minimum acceleration, acceleration variance, and lateral deviation from the lane center were included. All of these predictors were calculated for five segments before the conflict zone: 50–25 m, 25–20 m, 25–15 m, 25–10 m, and 15–10 m.

The dataset was divided into a training and a test set, with 70% of all trials used for training and the remaining 30% reserved for testing model performance. The split was performed at the subject level, ensuring that all trials of a given participant were assigned exclusively to either the training or the test set. This resulted in 22 participants (376 trials) in the training set and 9 participants (149 trials) in the test set. All subsequent preprocessing steps were conducted exclusively on the training data. To avoid redundancy, predictors with correlations greater than 0.90 were removed. Subsequently, feature selection using participant-grouped recursive feature elimination reduced the predictor set to 21 features.

Because the outcome variable (driving order) was imbalanced, the training data were oversampled within each cross-validation fold using the ROSE package to achieve a balanced distribution between the two outcome classes. Model tuning was performed using participant-grouped 5-fold cross-validation, repeated 10 times, and the optimal number of features randomly sampled at each split was selected automatically based on the best model performance using the package caret. All models were trained with 500 trees.

The best-performing model achieved an accuracy of 0.91 (95% CI [0.85, 0.95]), a balanced accuracy of 0.73, a sensitivity of 0.46 (95% CI [0.27, 0.67]), a specificity of 1.00 (95% CI [0.97, 1.00]), and a F1-Score of 0.63. The confusion matrix for the test set is shown in Table 4. Feature importance is shown in Table 5, with the relative distance to the priority vehicle 10 m before the intersection and speed and acceleration-related variables from the 25–10 m segment being the strongest predictors.

**Table 4:** Confusion matrix for the best-performing random forest model (test set).

Prediction	Actual	
	Participant drove first	Participant stopped
Participant drove first	12	0
Participant stopped	14	123

**Table 5:** Feature importance for the random forest model predicting driving order (scaled 0–100).

Feature	Importance
Relative distance between ego vehicle and priority vehicle at 10 m before the conflict zone	100.00
Speed variance in the 15–10 m segment	40.84
Speed variance in the 25–10 m segment	30.86
Minimum acceleration in the 25–10 m segment	28.13
Mean acceleration in the 25–10 m segment	28.00
Relative distance between ego vehicle and priority vehicle at 25 m before the conflict zone	20.03
Relative distance between ego vehicle and yielding vehicle at 10 m before the conflict zone	18.99
Maximum acceleration in the 50–25 m segment	18.36
Minimum acceleration in the 20–15 m segment	17.45
Maximum acceleration in the 25–10 m segment	16.12
Mean acceleration in the 15–10 m segment	15.33
Relative distance between ego vehicle and yielding vehicle at 25 m before the conflict zone	14.15
Approach position right	14.08
Speed variance in the 20–15 m segment	11.84
Variance in acceleration in the 25–20 m segment	11.19
Maximum acceleration in the 15–10 m segment	7.65
Variance in acceleration in the 15–10 m segment	4.04
Approach position below	1.81
Speed variance in the 25–20 m segment	1.22
Minimum speed in the 25–10 m segment	1.02

## 5 Discussion

The present study aimed to investigate driver behavior in deadlock situations at T-intersections, with a particular focus on how drivers approach such situations before a potential standstill occurs. The study examined whether drivers proceed first through the intersection without stopping beforehand or decide to stop (regardless of their subsequent crossing order), and how this decision is influenced by approach position and the arrival order of the two cooperating vehicles. In addition, the perceived ambiguity of the situation and drivers’ gaze behavior toward the cooperating vehicles were analyzed. Furthermore, driving dynamics during the approach to the intersection were examined, and it was assessed whether the driving behavior (proceeding first vs. stopping) can be predicted based on approach-related variables.

### 5.1 Driving order in deadlock situations

Across all situations, proceeding first (without stopping beforehand) was not the predominant response, but occurred in a substantial proportion of cases. The overall probability of driving first was comparable to previous video-based studies, which reported probabilities between 19% and 24% (Strelau and Deml, 2025, 2026). The slightly higher values reported in those studies can plausibly be attributed to methodological differences, as participants indicated intended behavior whereas the present study examined actual driving behavior in a simulator.

With regard to approach position, distinct patterns emerged across conditions. When approaching from the bottom, drivers almost exclusively stopped, replicating findings from previous video-based studies (Strelau and Deml, 2025). For the right approach, about 20% of drivers proceeded first without stopping beforehand, which aligns with earlier evidence showing that, under simultaneous arrival conditions, drivers from the right are more likely to proceed first than from other approach positions (Strelau and Deml, 2025). Arrival order influenced driving behavior only for the left approach. Specifically, when the vehicle that had priority relative to the ego vehicle arrived later at the intersection, drivers approaching from the left proceeded first without stopping beforehand in nearly half of the cases. Taken together, these findings indicate that temporal factors affect driving behavior primarily in combination with spatial factors, particularly for the left approach. Possible explanations for this pattern are discussed in section 5.4.

### 5.2 Perceived ambiguity and conflict interpretation

Arrival order did not affect perceived ambiguity or hesitation. Similarly, the approach position did not affect hesitation. Approach position had a significant effect on ambiguity, however, the effect explained only a small portion of the variance, and is therefore unlikely to be practically meaningful. Ambiguity and hesitation ratings also decreased between the first and second run through the intersections, potentially because participants became more familiar with the recurring traffic configurations. Moreover, no differences in perceived ambiguity emerged between drivers who proceeded first without stopping beforehand and those who stopped (regardless of their subsequent crossing behavior). Across all trials, the situation was rated as low to moderate in ambiguity, and drivers indicated low hesitation regarding their behavioral decision, suggesting that drivers were fairly confident in their interpretation of the right-of-way situation. Previous work (Strelau and Deml, 2026) showed that most drivers assume that one of the three vehicles has priority and that drivers who believe another vehicle has priority tend to stop. However, it remained unclear whether stopping behavior reflects uncertainty and cautious decision-making or a confident interpretation of right-of-way in favor of another vehicle. The present findings support the latter interpretation: stopping behavior appears to be based on a fairly confident, though not always correct, assessment of right-of-way rather than on uncertainty. From a space-sharing conflict perspective, this implies that the situation is not necessarily experienced as a conflict. If drivers assume that one vehicle clearly has the right-of-way, they do not anticipate competing space claims and therefore do not represent the situation as a potential space-sharing conflict. Stopping behavior in this case does not reflect interactive behavior to resolve the conflict, but rather a straightforward application of an assumed priority rule.

### 5.3 Gaze behavior during the approach

Fixations do not necessarily indicate that the corresponding information was fully processed (Herslund and Jørgensen, 2003), and information can also be perceived through peripheral vision without direct fixation (Wolfe et al., 2022). Nonetheless, fixations have been shown to increase the likelihood of subsequent processing (Underwood et al., 2003), and because fixations indicate where visual attention is directed, they are a widely used measure of attention allocation in the driving literature (Kircher and Ahlström, 2020; Lemonnier et al., 2015; Robbins and Chapman, 2018). Gaze behavior during the approach to the deadlock situation can provide a potential explanation for why drivers report relatively low ambiguity despite being in an objectively ambiguous deadlock situation. While the ambiguity ratings suggest that drivers do not experience the situation as a conflict and form fairly confident right-of-way interpretations, gaze data suggest that this confidence could be associated with incomplete visual scanning of the scene. In many cases, at least one of the cooperating vehicles was not fixated or not visible during the approach phase. By failing to explicitly attend to all cooperating vehicles, drivers may reduce the complexity of the situation. What is formally a triadic deadlock may be experienced as a simpler two-vehicle interaction with a clear right-of-way allocation. It is plausible to speculate that the tendency toward clear right-of-way interpretations in these cases is not formed despite incomplete scanning, but rather as a consequence of it, although direct causal evidence remains to be established. In particular, the vehicle that has to yield to the ego vehicle was significantly more often not fixated or not visible than the vehicle with priority relative to the ego vehicle. While drivers may still register such vehicles via peripheral vision or brief glimpses that were not captured by the fixation measure, the absence of fixations nevertheless suggests a reduced likelihood of detailed processing. Not fixating the yielding vehicle may therefore be associated with a reduced likelihood that drivers integrated its status into their mental model of the situation and identified the situation as a deadlock at all. This selective perception may then reinforce drivers’ initial right-of-way interpretation.

Gaze behavior depended on both assumed right-of-way and approach position. While drivers approaching from the left fixated the yielding vehicle in approximately half of the cases, fixation rates dropped to about 25% or less for approaches from the right and bottom. For the vehicle with priority relative to the ego vehicle, fixation rates were generally high, but lower for left and bottom approaches than for the right approach. These differences are consistent with differences in visual accessibility: vehicles arriving from the side, requiring head rotation, were fixated less frequently than vehicles opposite of the driver. Cases in which a vehicle was not visible at all occurred exclusively for vehicles requiring head movement. In addition, the yielding vehicle was even less frequently fixated or visible when it arrived later at the intersection compared to conditions with simultaneous arrival or when the priority vehicle arrived later.

These findings are consistent with results reported by Strelau and Deml (2026), who showed that the degree of accurately identifying the travel direction of the cooperating vehicles was dependent on approach position and their relative priority. They also align with results by Werneke and Vollrath (2014) demonstrating that drivers preferentially attend to vehicles they have to yield to, even when other relevant road users are present. The observed allocation of visual attention can be interpreted within the SEEV model (Wickens et al., 2003), which describes attention as a function of salience, effort, expectancy, and value of information.

In the present study, the influence of value is consistent with drivers predominantly fixating the vehicle with priority relative to the ego vehicle, indicating that this vehicle was treated as the most informative for guiding behavior. At the same time, salience played a critical role: vehicles approaching from the side and requiring head rotation were fixated less frequently than vehicles directly in front of the driver. The joint influence of value and salience becomes particularly apparent for the left approach. Here, the yielding vehicle was fixated substantially more often than for the right approach, despite having no priority relative to the ego vehicle. This increased fixation rate cannot be explained by value, but is consistent with its high visual salience, as the vehicle approached from directly ahead. In contrast, the vehicle with priority relative to the ego vehicle was fixated less frequently during the left approach than during the right approach. Together with the pattern observed for the yielding vehicle, this suggests an interaction between reduced salience and subjective value assignment. When approaching from the left, some drivers appear to assume that they themselves have priority. This assumption was previously observed in deadlock situations (Strelau and Deml, 2026) and is consistent with the tendency to attribute priority to the straight road (Björklund and Åberg, 2005). Under this assumption drivers may have attributed lower informational value to this vehicle and therefore allocated less attention to it. The effect is further reinforced when this vehicle arrives later at the intersection when its salience is reduced. Together, these results are consistent with both top-down (value) and bottom-up (salience) factors contributing to drivers’ visual attention in deadlock situations, resulting in selective attention patterns that may prevent drivers from fully integrating all vehicles involved in the deadlock situation.

It should be noted that the findings are specific to the analyzed approach segment between 25 and 10 m before the conflict zone. An earlier starting point would likely not have altered the reported gaze patterns towards the vehicle requiring head rotation, as this vehicle may not yet have been visible at that point. A later end point, in contrast, would possibly have shown increasing fixation rates towards both cooperation vehicles, simply because participants had more time to scan the scene as they approached the intersection. However, by this later stage, a substantial proportion of participants had already come to a stop, meaning that gaze behavior would no longer reflect the approach phase but rather behavior following an initial stopping decision. The present findings should therefore be understood as characterizing gaze allocation specifically during the approach phase, rather than across the full interaction with the cooperation vehicles.

### 5.4 Driving order in relation to gaze behavior and driving dynamics

The relationship between gaze behavior, driving dynamics, and driving order differed across approach positions. For the right approach, no significant differences in gaze behavior were observed between drivers who proceeded first without stopping beforehand and those who stopped (regardless of their subsequent crossing behavior). This is consistent with the generally high fixation rates for the priority vehicle at this approach position. As this vehicle is visible throughout the whole approach to the intersection and drivers seem to attribute more relevance to this vehicle compared to the yielding vehicle, it may already be treated as the most relevant vehicle regardless of arrival order, leaving comparatively little room for arrival order to further affect gaze behavior. This, in turn, may explain why arrival order did not appear to affect driving order for the right approach, possibly because any underlying right-of-way interpretation remained unaffected by the timing of the priority vehicle’s arrival, unlike for the left approach, discussed below. Similarly, no significant differences in driving dynamics, including mean speed, speed variance and acceleration, were found between drivers who proceeded first and those who stopped, nor between the arrival orders for the right approach. This absence of differences in driving dynamics should be interpreted with some caution, as all drivers approaching from the right subsequently turned at the intersection, which likely required deceleration irrespective of driving order or arrival order, and may have masked differences that could otherwise reflect differing assumptions about right-of-way. Overall, proceeding first from the right was not associated with a distinct perceptual or dynamic approach pattern.

A different pattern emerged for approaches from the left. Here, significant differences were observed both in gaze behavior and in driving dynamics between drivers who proceeded first without stopping beforehand and those who stopped (regardless of their subsequent crossing behavior). Drivers who proceeded first frequently did not fixate the vehicle with priority relative to the ego vehicle, with this vehicle not being fixated in nearly half of the cases. In contrast, among drivers who stopped, this vehicle was not fixated in only about a quarter of the cases. The opposite pattern was observed for the yielding vehicle, which was fixated more often by drivers who proceeded first.

These differences in gaze behavior were accompanied by systematic differences in the approach to the intersection. While no differences in mean speed during the approach were found, drivers who drove first through the intersection showed significantly lower variance of speed and less deceleration compared to drivers who stopped. This pattern suggests that these drivers did not prepare to yield but instead approached the intersection in a manner consistent with the assumption that they had priority.

Taken together, the convergence of driving dynamics and gaze behavior suggests that for the left approach, the decision to proceed first may not have been a reactive response to the behavior of the other vehicles, but could instead reflect an early and consistent assumption of priority shaped by both visual attention and driving behavior throughout the approach. This may help explain why arrival order affected driving order specifically for the left approach. When the priority vehicle arrives later, and therefore becomes visible only later during the approach, it is less frequently fixated and the assumption of having priority may be more likely to remain uncorrected.

Interestingly, a similar pattern in driving dynamics emerged when comparing arrival order conditions among drivers who stopped. When the vehicle with priority relative to the ego arrived later, even drivers who ultimately stopped showed lower variance of speed and less deceleration compared to conditions in which the vehicle that has to yield to the ego arrived later or both cooperating vehicles arrived simultaneously. Notably, drivers who proceeded first without stopping occurred dominantly in the priority-over-ego-later condition. However, even within this condition, they still showed lower variance of speed and less deceleration than those who stopped. This suggests that the arrival order of the cooperating vehicles shapes initial approach behavior of all drivers, irrespective of their eventual decision. When the priority vehicle arrives later, drivers generally approach the intersection in a manner less consistent with yielding. However, drivers who ultimately proceed first exhibit this pattern to an even greater extent. One possible explanation for these patterns is that the priority vehicle may have been detected relatively late in these situations. As most drivers who stopped did fixate the priority vehicle, it is conceivable that this fixation and thus recognition of the need to yield occurred only at a later stage of the approach (e.g., closer to the 10 m threshold used for the analysis). Because the timing of first fixation was not analyzed, this possibility cannot be ruled out. However, even under this assumption, the observed driving pattern still suggests that drivers did not initially anticipate the need to yield, as they showed less preparatory behavior, e.g., less deceleration compared to the other conditions.

The interpretations offered above link gaze behavior and driving decisions to an assumed right-of-way interpretation, but alternative explanations for the observed patterns cannot be ruled out, for instance habitual scanning patterns or behavior based on former experiences. Consistent with this, the gap between the marginal and conditional R^2^ across the reported models indicates that a part of the variance is explained by stable differences between participants, which could reflect either such general behavioral tendencies or, alternatively, stable differences in right-of-way interpretation itself.

### 5.5 Predicting driving order from approach-related behavior

To assess whether drivers’ decisions to proceed first without stopping beforehand or to stop (regardless of their subsequent crossing behavior) can be predicted while approaching the intersection, a random forest classifier was trained using driving parameters and the relative distance to the cooperating vehicle, all extracted from the approach segments between 50 m and 10 m before the conflict zone. Importantly, only variables that can be observed from outside the vehicle were included, making the prediction approach directly applicable to automated vehicles.

Overall, the model achieved a balanced accuracy of 0.75. Prediction performance differed between the two outcomes. Stopping behavior was identified with very high accuracy, whereas proceeding first was predicted with a lower sensitivity. This asymmetry is likely related, at least in part, to the unequal frequency of the two behaviors in the dataset, with only 17% of trials involving participants who proceeded first, providing fewer observations from which the classifier could learn the characteristics of this behavior. Although oversampling was used to reduce the influence of class imbalance during model training, this cannot fully compensate for the limited amount of empirical information available for the minority class. Thus, the high overall accuracy of 0.91 should be interpreted with caution, as it is partly driven by the model’s high performance in identifying the more frequent stopping behavior. Larger and more balanced samples may therefore improve the prediction of proceeding-first behavior.

A potential limitation is the risk of overfitting given the relatively small sample size. This risk was reduced by splitting the data at the subject level, so that no participant contributed trials to both the training and test sets, and by using participant-grouped 5-fold cross-validation for model training. Nevertheless, the relatively small number of participants means that overfitting cannot be completely ruled out, and the observed predictive performance may not fully generalize to independent samples. Additionally, the reported sensitivity of 0.46 corresponds to only 12 of 26 correctly identified proceeding-first trials and relies on a single train-test split with only 9 participants in the test set. These estimates should therefore be interpreted with caution.

The results indicate that driving behavior in deadlock situations can, in principle, be predicted before the intersection is reached, although prediction accuracy for drivers who proceeded first without stopping beforehand remained limited. Additionally, it should be noted that the predictors used are temporally close to the outcome itself, as some features were extracted from the segment up to 10 m before the conflict zone, at a point where drivers may have already begun to brake. The model therefore does not predict driving behavior far in advance, but rather estimates drivers’ likely intentions in the final meters of the approach. In this sense, the present analysis is best understood as a demonstration of principle: it shows that the approach phase contains information about subsequent driving order, even if the model is not yet suitable for direct implementation in autonomous vehicles.

Incorporating additional information closer to the conflict zone may further improve prediction performance. Moreover, temporal relations between vehicles offer additional potential for refinement. Arrival order in the present study differentiated only between simultaneous and later arrival. In real traffic, arrival-time differences are likely to vary more continuously. Such graded temporal information may provide additional predictive cues and further enhance the prediction of driving behavior.

### 5.6 Implications for autonomous vehicles

The present findings provide guidance for AVs in deadlock situations at T-intersections, extending and refining recommendations from previous studies (Strelau and Deml, 2025, 2026). Drivers approaching from the bottom almost always stopped, confirming earlier observations. This suggests that an AV in this position should adopt a defensive strategy and stop. When all vehicles arrive approximately simultaneously, drivers from the left also mostly stop, providing an AV approaching from the right with the best opportunity to proceed first. However, if the vehicle from below arrives later, roughly half of drivers approaching from the left attempt to proceed first without stopping beforehand. In this case, the right approach may no longer be the optimal position to drive first, and an AV approaching from the left could potentially take the initiative. This would revise previous recommendations (Strelau and Deml, 2026), which suggested that the left approach is generally unsuitable for driving first because drivers do not expect to have priority here. The present data indicate that when the vehicle from below arrives later, drivers approaching from the left often assume to have priority and may fail to recognize a potential conflict, which could make this position suitable for an AV to drive first. However, this interpretation would benefit from further empirical testing.

Similarly, the general claim that drivers in deadlock situations at T-intersections prefer another vehicle to drive first does not hold universally. Both approach position and arrival order matter, as there are conditions in which drivers themselves proceed first. For this reason, it is valuable for an AV to be able to anticipate whether the other vehicles will stop. As a proof of concept, the present study demonstrates that driving order (i.e., whether a driver proceeds first without stopping beforehand or not) can, in principle, be predicted based on externally observable factors, achieving a balanced accuracy of approximately 0.73. The present model should be understood as a first attempt to demonstrate this feasibility, rather than a model ready for deployment, as sensitivity for correctly identifying drivers who proceed first remained low (0.46). With a more refined model, such predictions could in the future allow an AV to estimate the likelihood that the other vehicle will stop. Moreover, the AV could reinforce a yielding expectation by communicating its intent through slightly more assertive behavior, which has been shown to influence drivers’ yielding decisions (Imbsweiler et al., 2018).

Combining prediction of driving order with intention communication, and adapting behavior based on approach position and arrival order, could in principle help an AV resolve deadlocks more effectively and reduce the risk of collisions or standstills. However, these recommendations are derived from observations of human driver behavior rather than from testing an AV in these situations, and further research would be needed before translating these findings into concrete design recommendations. Nonetheless, by identifying the specific conditions under which human drivers proceed first, stop, or fail to fully recognize a deadlock, the present findings offer a starting point for developing AV strategies that remain aligned with human drivers’ expectations.

### 5.7 Limitations

Several limitations of the present study should be acknowledged. The study was conducted in a driving simulator rather than in real traffic, and findings obtained under simulated conditions may not fully represent real-world driving behavior. Although driving simulators are well established for investigating driver behavior, and many aspects transfer reliably to real-world settings (Robbins et al., 2019), differences between these two may have nevertheless influenced drivers’ behavior. Gaze analyses were limited to fixations on the two cooperating vehicles and to the approach segment between 25 m and 10 m before the conflict zone. Peripheral perception without direct fixation on the vehicles and visual behavior closer to the intersection were not captured. Consequently, conclusions regarding gaze behavior and perception of the deadlock situation apply only to this earlier approach phase.

The final sample size was relatively small due to a substantial dropout rate. This may have reduced the statistical power required to detect smaller effects, particularly interaction effects, and non-significant findings should therefore be interpreted with caution. The sample predominantly consisted of young male drivers, possibly because female participants are more likely to discontinue participation due to simulator sickness (Matas et al., 2015). While this is a plausible contributing factor, other unmeasured differences between those who completed the study and those who dropped out cannot be entirely excluded. As age and gender have been shown to be associated with differences in driving behavior and visual scanning behavior (Bao and Boyle, 2009; Yan et al., 2007), the present findings may not be generalizable to the broader driving population. The findings may also not be generalizable to different road types and speed zones, as the study was conducted in a 30 km/h zone in an urban environment. Deadlock behavior may differ under higher speed limits, such as in a 50 km/h zone, due to shorter available reaction times, for example.

Finally, the cooperating vehicles only showed defensive behavior. As drivers adapt their own behavior to that of other road users (Imbsweiler et al., 2018), it is plausible that more offensive behavior by a cooperating vehicle would alter drivers’ gaze behavior, approach dynamics, perceived ambiguity and decision to drive first or stop. This is particularly intriguing given that many drivers appear to not experience the deadlock situation as a conflict. Future studies should therefore examine how the interaction of arrival order and different behavioral strategies of cooperating vehicles influence drivers’ perception and behavior in deadlock situations.

## 6 Conclusion

The present study examined how drivers approach and resolve deadlock situations at T-intersections before a potential standstill occurs. The results show that drivers generally do not experience these situations as particularly ambiguous. Instead, they appear to form early and fairly confident interpretations of right-of-way, which appear to be reflected in their visual attention, approach behavior, and decision to proceed first or to stop. This finding supports and extends previous work (Strelau and Deml, 2026), demonstrating that many drivers do not correctly apply formal right-of-way rules but assume that one of the vehicles has priority. Gaze behavior further revealed that drivers often failed to fixate all cooperating vehicles, particularly those requiring head rotation or that have to yield to them. This incomplete scanning may itself explain why drivers tend to report relatively low ambiguity ratings and appear to not experience the situations as a conflict: by not attending to all vehicles involved, the triadic deadlock may effectively be reduced to a simpler two-vehicle interaction, which in turn could reinforce drivers’ initial interpretation of right-of-way. Driving behavior was shaped by both approach position and arrival order. Most notably, for the left approach, a coherent pattern emerged across gaze behavior and driving dynamics. Drivers who proceeded first without stopping beforehand less frequently fixated the priority vehicle, showed lower speed variance and less deceleration, and did so predominantly when the priority vehicle arrived later, indicating that approach position alone is insufficient to explain driving decisions in deadlock situations. Both gaze behavior and driving dynamics thus are consistent with an early and consistent assumption of priority rather than a purely reactive response. Finally, as a proof of concept, the study demonstrates that drivers’ decisions can, in principle, be predicted from observable variables before the intersection is reached, although prediction accuracy for drivers who proceeded first remained limited. Combined with the behavioral patterns identified in this study, these findings provide a basis for anticipating human drivers’ behavior and offer a starting point for deriving recommendations for autonomous vehicles that could help adopt strategies that efficiently resolve deadlocks while remaining aligned with human expectations.

## Data Availability

The datasets presented in this article are not readily available because the informed consent obtained from participants only permits the publication of aggregated data and results. Aggregated data supporting the conclusions of this article will be made available by the authors, without undue reservation. Requests to access the datasets should be directed to nadine-rebecca.strelau@kit.edu.
